# Supplementary material for: A molecular map of murine lymph node blood vascular endothelium at single cell resolution
Source: Nat Commun. 2020 Jul 30;11:3798. doi: 10.1038/s41467-020-17291-5 (PMC7393069; doi:10.1038/s41467-020-17291-5)
Supplement: Supplementary file 1 — Supplementary Information [file 41467_2020_17291_MOESM1_ESM.pdf]

Supplementary Information

Brulois et al., A molecular map of murine lymph node blood vascular endothelium at single cell resolution

Supplementary Figures 1-10

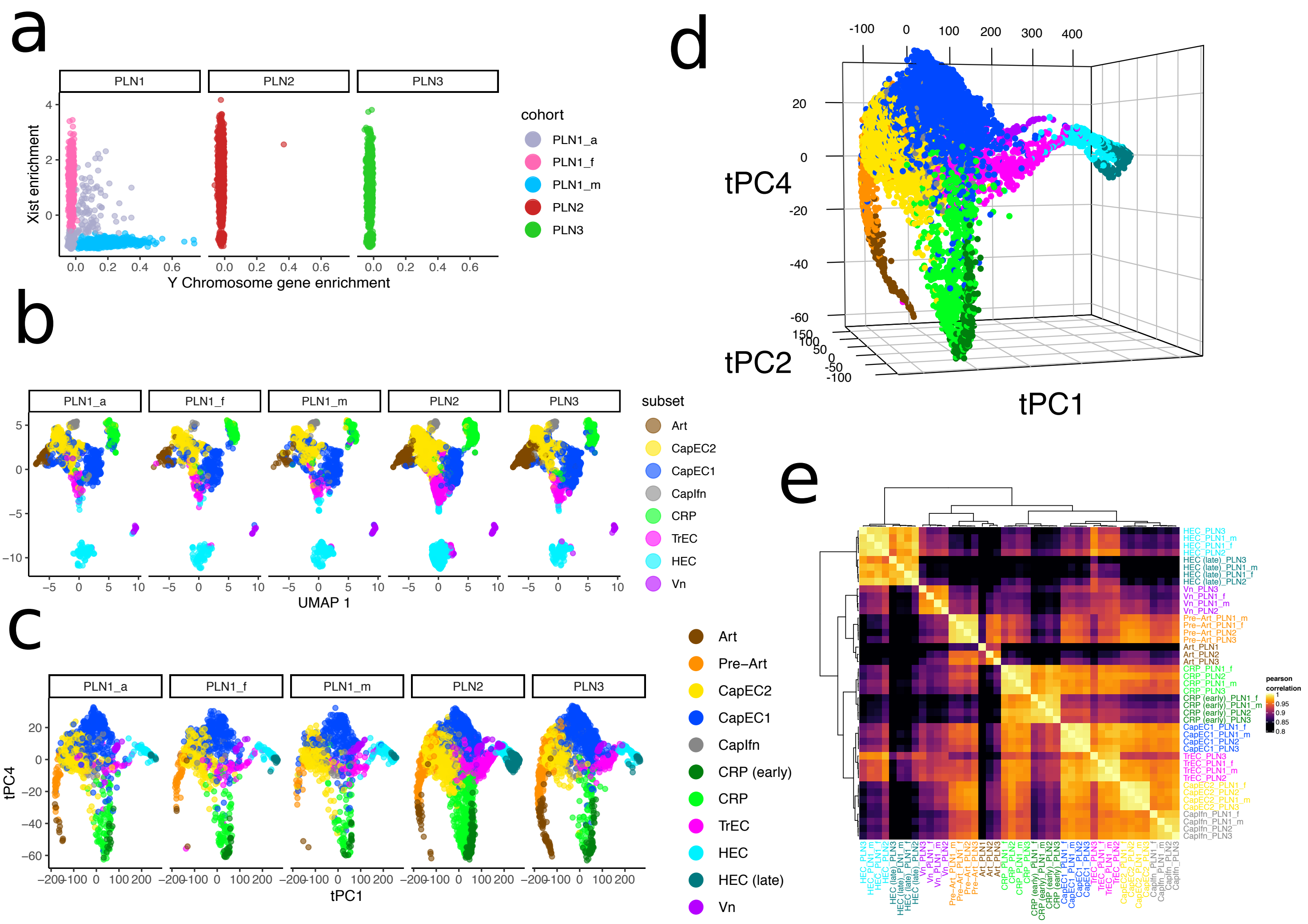

Supplementary Figure 1 | Consistency of three technical replicates and four independent mouse cohorts.

(a) Scatter plots of all cells showing separation of the PLN1 sample into male and female cohorts (PLN1_m and PLN_f) and remaining unclassifiable PLN1_a cells. (b) UMAP plot from Fig. 1d, stratified by cohort. (c) and (d) tSpace projection (2D in (c) and 3D in (d)) using all cells colored by subset. Interactive rendering available: https://stanford.io/2WXR811 (e) Pearson correlation of gene expression profiles of subsets from different cohorts. A set of ~2000 differentially expressed genes was used to calculate mean expression profiles for each of the major subsets (Art, CapEC2, CapEC1, CapIfn, CRP, TrEC, HEC, Vn) in each cohort (PLN1_m, PLN1_f, PLN2 and PLN3). Cells of the Art subset from the PLN1_m and PLN_f cohorts were combined and treated as a single cohort due to low total number of Art cells in PLN1_m. Expression profiles were hierarchically clustered and plotted along with their pairwise Pearson correlation coefficients (color scale).

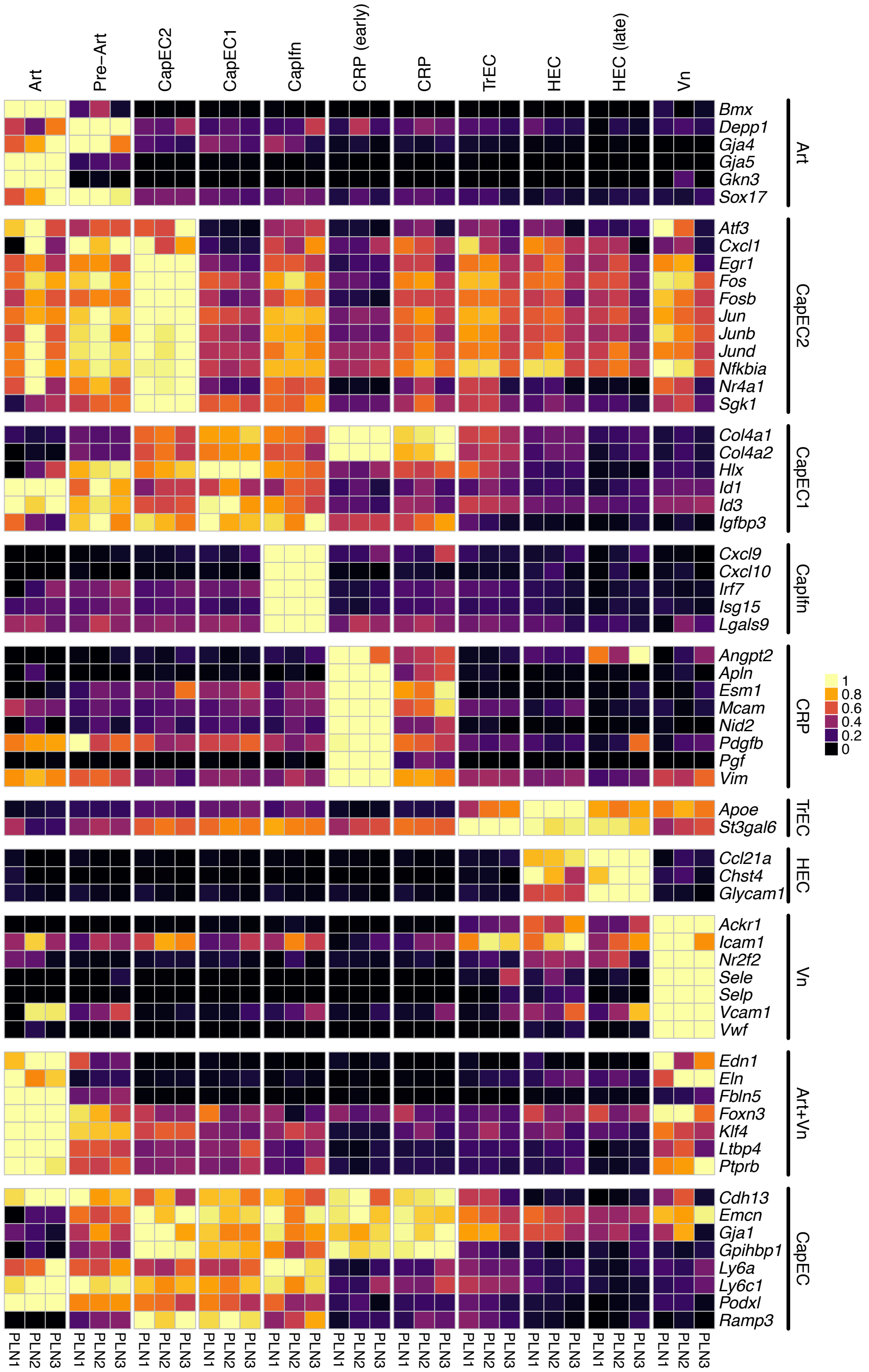

Supplementary Figure 2 | Consistency of gene signatures from Fig 1 across 3 replicates.

Average expression of genes shown in Figure 1f. Log-transformed normalized expression values scaled by dividing by the max on a per sample basis.

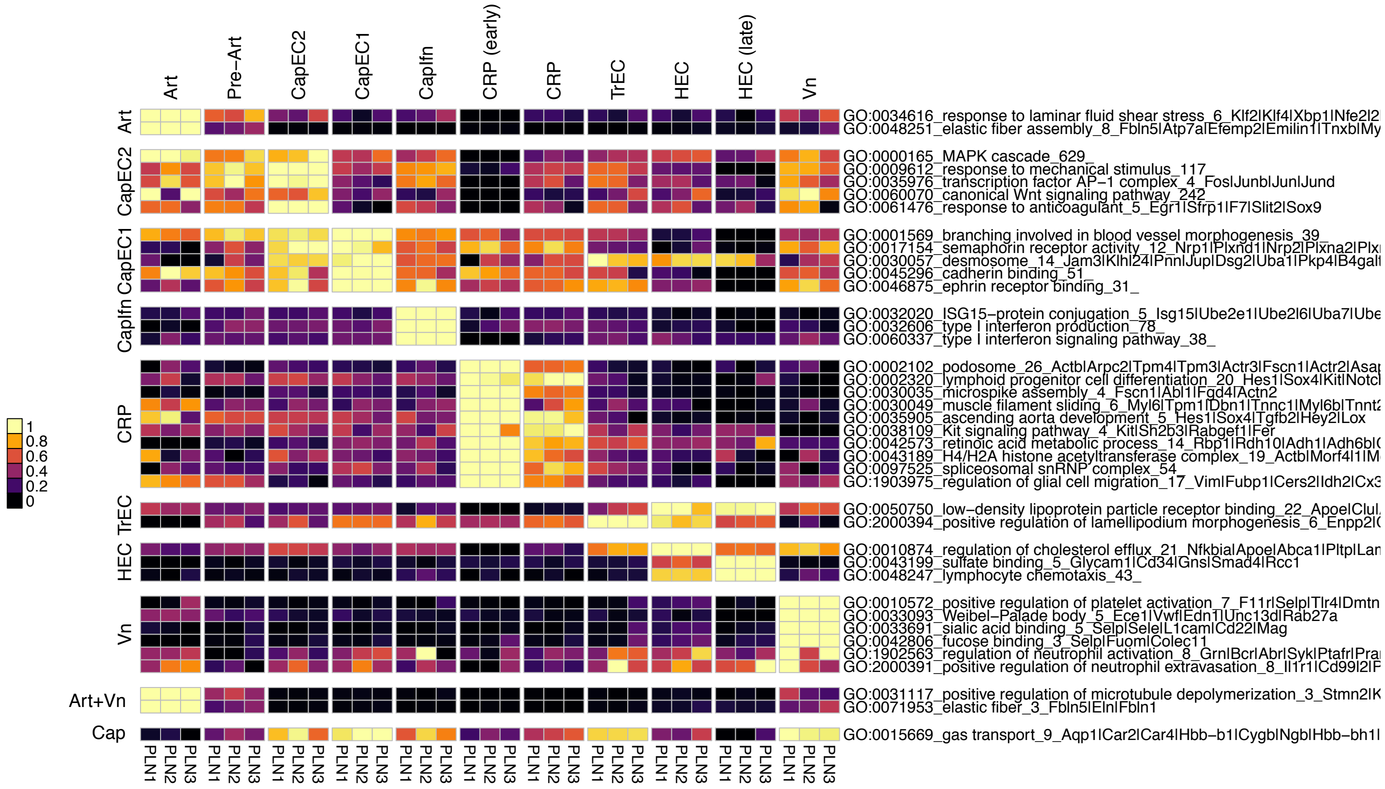

Supplementary Figure 3 | Pooled expression of genes grouped according to their Gene Ontology (GO terms).

Average pooled expression for select differentially enriched GO terms in each sample. The number of genes belonging to a given term is indicated after the term identifiers. When feasible, symbols for individual genes (separated by a “|”) belonging to given GO term are listed in order of highest to lowest expression across all datasets.

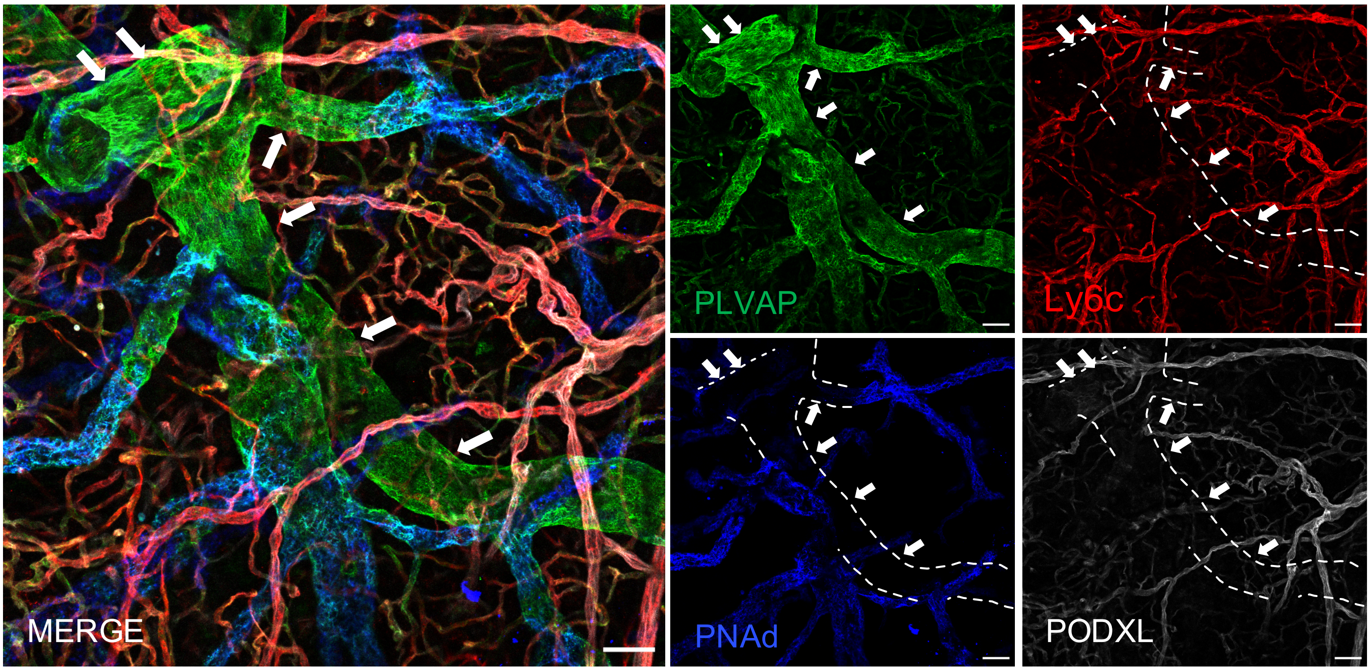

Supplementary Figure 4 | Additional image illustrating the medullary vein subset

Immunofluorescent image of PLN stained with i.v. injected anti-PLVAP (green), anti-Ly6c (red), anti-PNAd (blue) and anti-PODXL (white). Images representative of 3 independent experiments. Scale bar 50 μm. Arrows point to medullary vein.

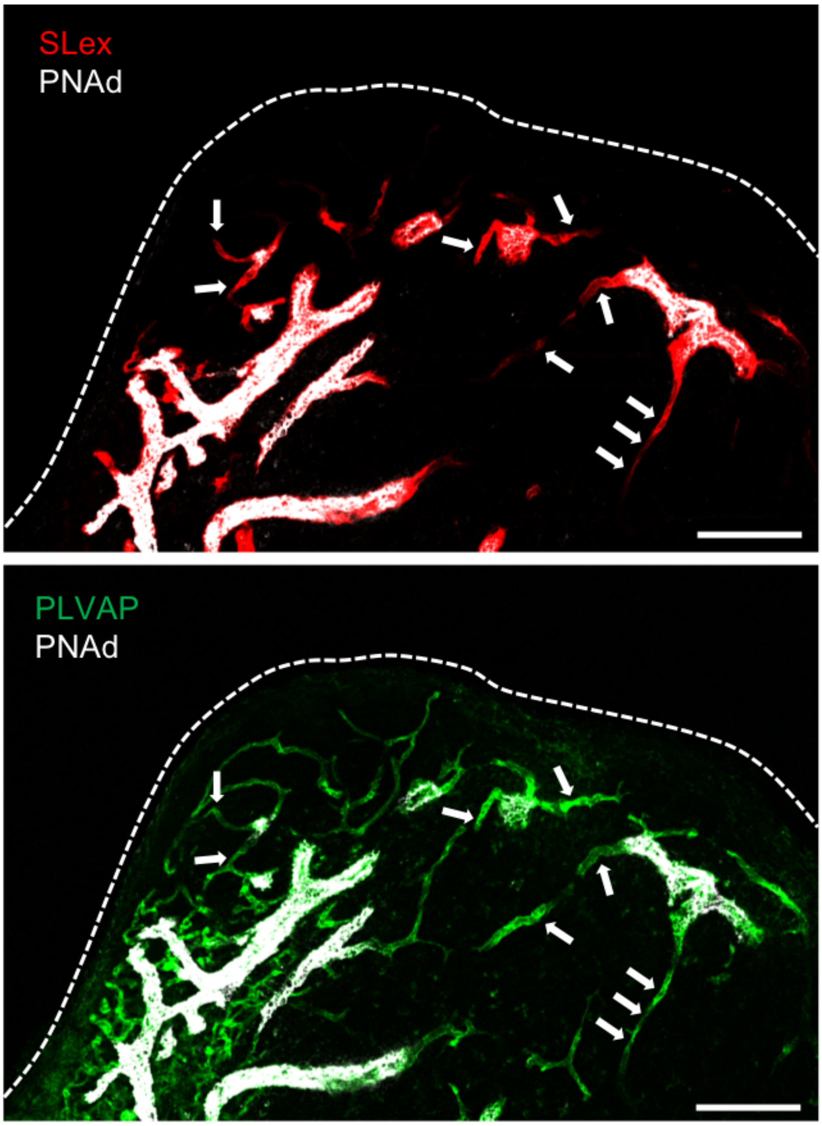

Supplementary Figure 5 | TrEC localize to capillary segments entering HEV.

Immunofluorescent image of PLN stained with i.v. injected anti-sLex (red), anti-PNAd (white) and anti-PLVAP (green). Arrows indicate TrEC, capillary segments expressing sLex but not PNAd. Images representative of 3 independent experiments. Bars, 100 µm. Dashed line, LN capsule.

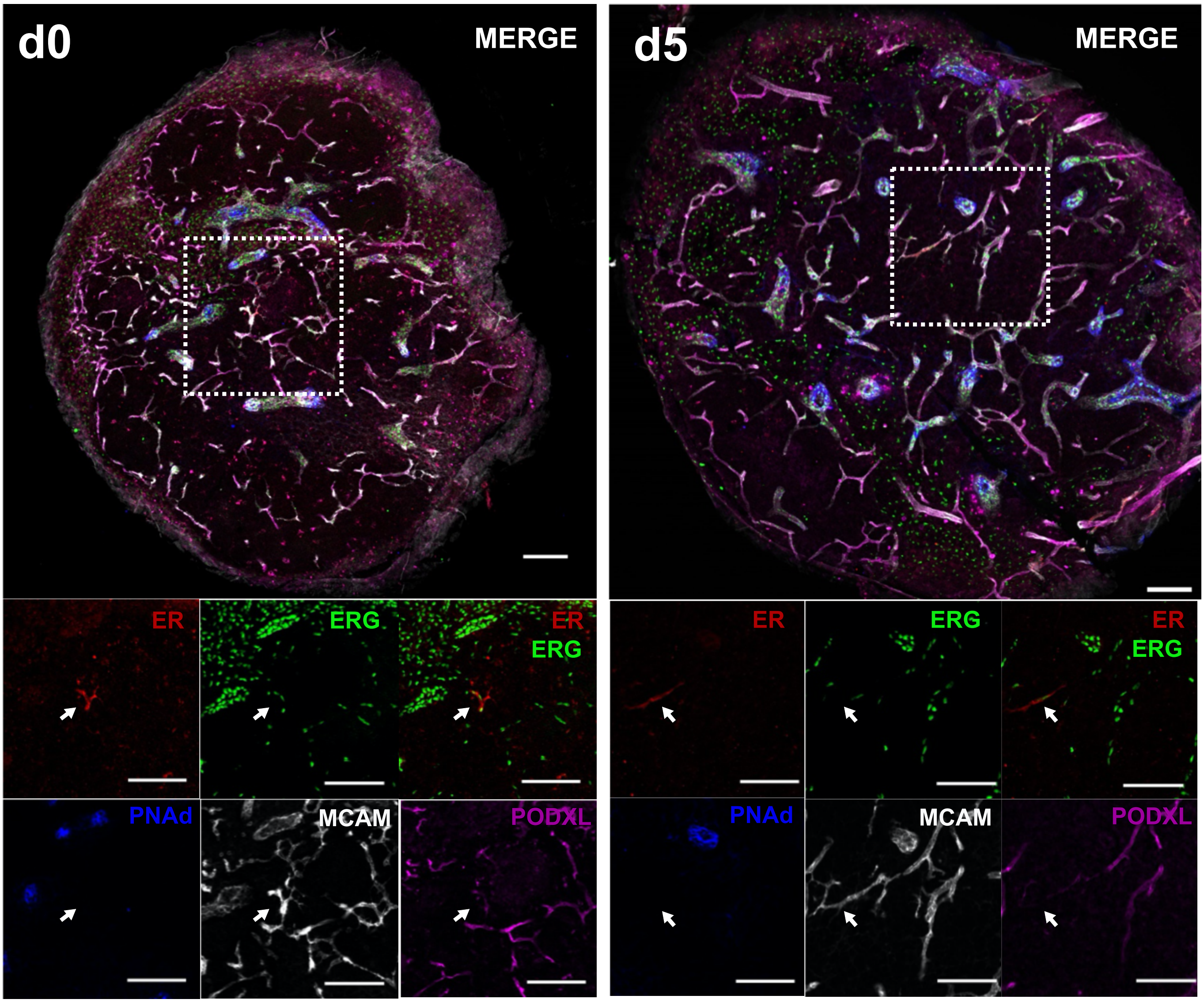

Supplementary Figure 6 | Additional markers characterizing the ER+ CRP subset

Immunofluorescent images of PLN from untreated mice (left) and mice treated with oxazolone for 5 days (right). PLN were stained with anti-ER (red), anti-ERG (green), anti-PNAd (blue), anti-MCAM (white) and anti-PODXL (violet). Images representative of at least 3 experiments. Scale bar 100 µm. Arrows point to CRP.

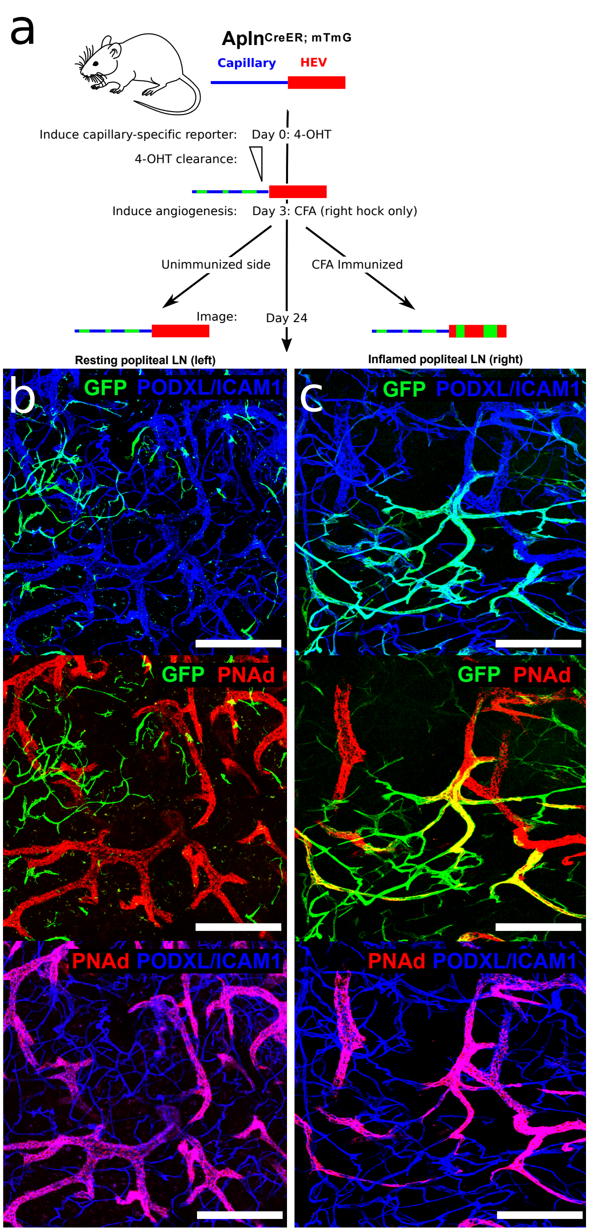

Supplementary Figure 7 | Lineage tracing of AplnERTCre-expressing capillary EC with additional 4-OHT clearance time.

(a) Experimental timeline for (b) and (c). Reporter expression was induced in Apln-CreER-mTmG mice by i.p. injection of 4-OHT. 72 hours later CFA was injected into the right hock and three and half week later mice were sacrificed. EC subsets were labeled by i.v. injection of the indicated antibodies 10-20 minutes before sacrifice.: anti-PNAd (red), anti-PODXL (blue) and anti-ICAM1 (blue). Representative images of resting (b) and inflamed (c) popliteal lymph nodes from 3 independent experiments. False color used to represent fluorophores. tdTomato not shown. Bars, 200 μm.

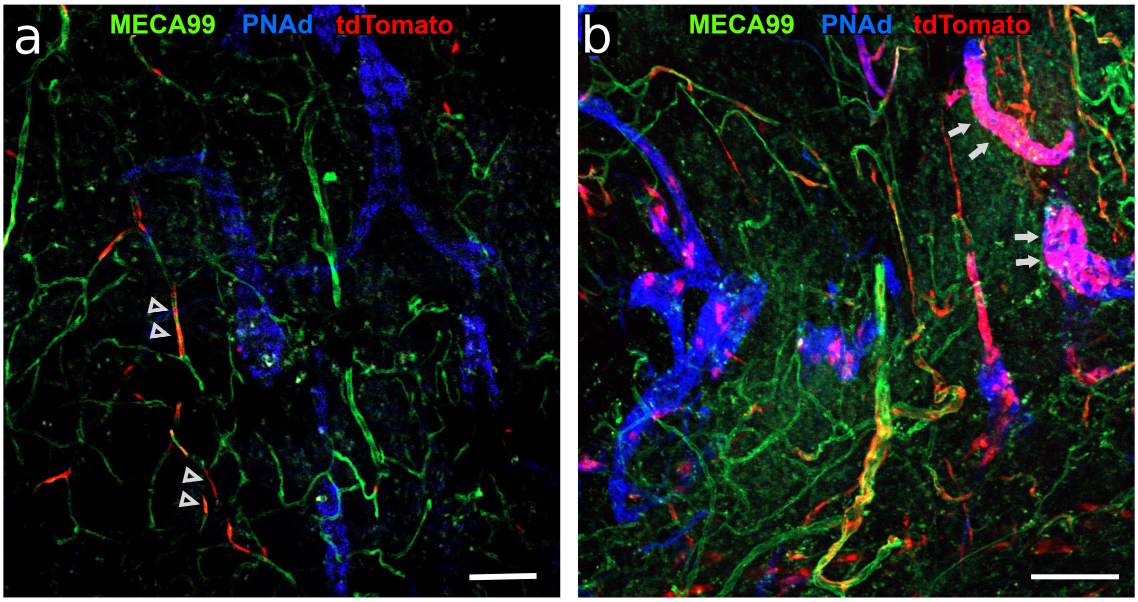

Supplementary Figure 8 | Tamoxifen administration during the early oxazolone response: selective capillary reporter induction and lineage tracing

Apln-CreERT2-tdTomato mice were immunized by cutaneous application of oxazolone, pulsed with i.p. tamoxifen the next day, and sacrificed either 48 hours (a) or twelve days (b) after immunization. EC subsets were labeled by i.v. injection of the indicated antibodies 10-20 minutes before sacrifice, and draining lymph nodes were imaged: PODXL (MECA99; green), PNAd (blue), tdTomato (red). Representative images of reporter (tdTomato) positive EC 48 hours (left; n = 3) or twelve days (right; n = 1) after immunization. Bars, 100 μm.

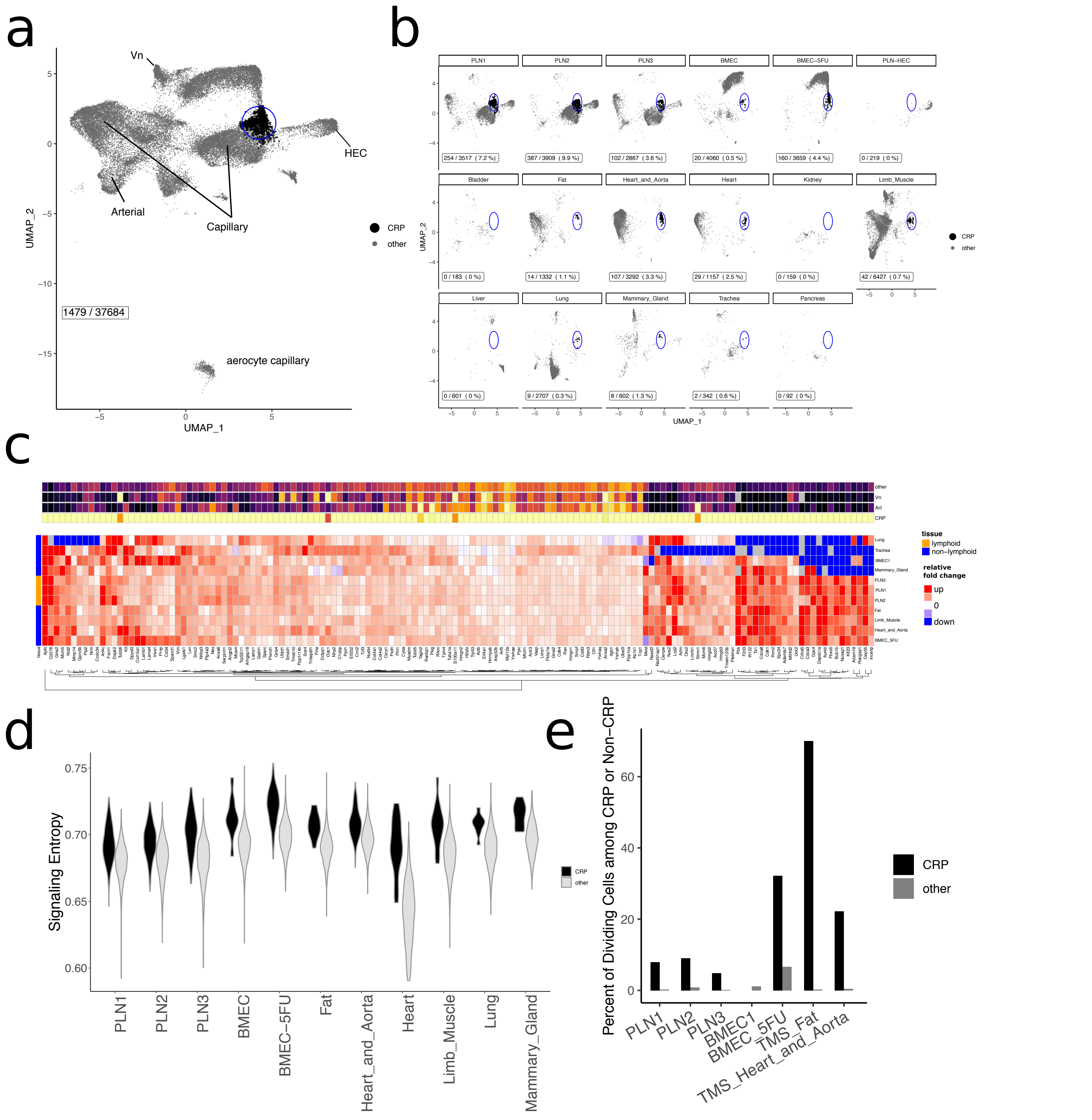

Supplementary Figure 9 | Global alignment of PLN BEC with publicly available single cell BEC profiles

(a and b) UMAP plot of MNN-aligned data from various public BEC datasets. a) all BEC aligned, illustrating the position of CRP-like EC. b) Separate plots for each tissue. Blue circle highlights the region to which LN CRP map. Black dots represent cells that align with LN CRP and that also are more similar in gene expression profile to CRP than to other LN EC subsets. PLN samples are from this study. PLN-HEC are sorted EC from mice. BMEC1 and BMEC_5FU are sorted Cdh5-reporter-positive EC from bone marrow. Other samples are from Tabula Muris consortium. (c) Heat maps of genes selectively expressed by LN CRP and CRP-like EC in other tissues. Top panel shows selectivity compared with other EC subsets (combined data all tissues). Bottom panel shows fold-change analysis of CRP-like EC compared to non-CRP in different tissues, and illustrates shared expression. (d) Violin plots of the signaling entropy rate of the indicated samples. (e) Bar graph depicting the percentage of CRP or of non-CRP that express signatures of cell division in each of the indicated samples (4 tissues, 7 independent samples).

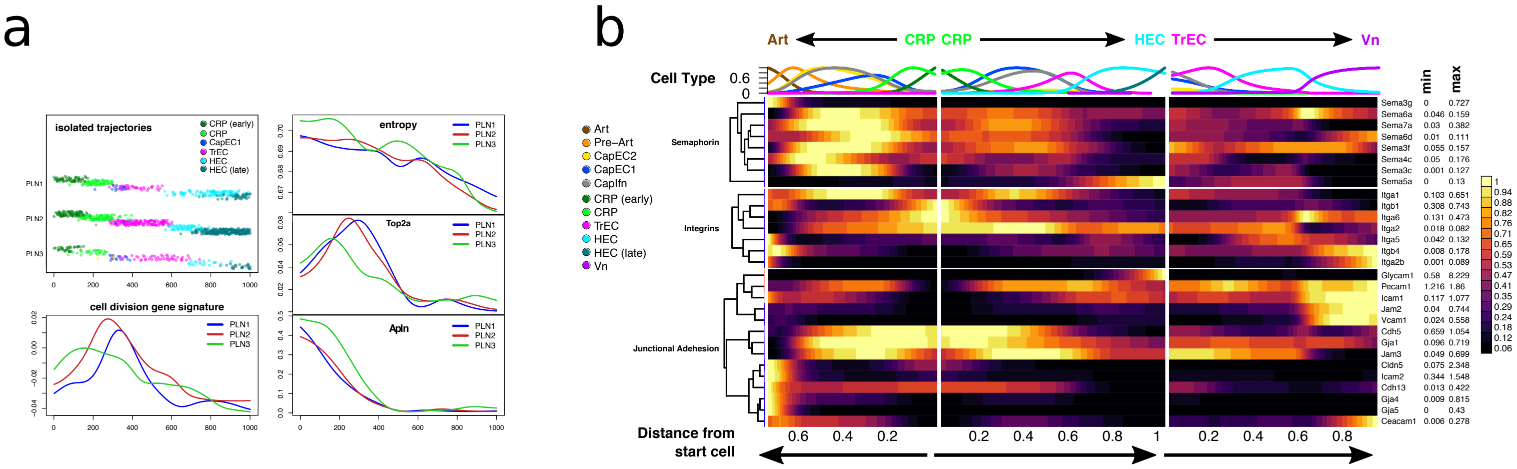

Supplementary Figure 10 | Expression of additional genes and features along isolated trajectories.

(a) Expression of select genes and features plotted along cell trajectories from early CRP to HEC and smoothed as in Figure 7. Apln is downregulated rapidly from early to late CRP, while markers of cell cycle (e.g. Top2a, and a global division signature), increase and peak in late CRP and TrEC. The “cell division gene signature” was quantified as a pooled expression value of previously defined cell division genes83. (b) Expression of selected genes along cell trajectories from early CRP to Art (plotted leftward), and from CRP to HEC or Vn (rightward) as in Figure 7.
