# Supplementary material for: A molecular map of murine lymph node blood vascular endothelium at single cell resolution
Source: Nat Commun. 2020 Jul 30;11:3798. doi: 10.1038/s41467-020-17291-5 (PMC7393069; doi:10.1038/s41467-020-17291-5)
Supplement: Supplementary file 4 — Reporting Summary [file 41467_2020_17291_MOESM4_ESM.pdf]

nature research

Corresponding author(s): Eugene Butcher

Last updated by author(s): March 13, 2020

Reporting Summary

Nature Research wishes to improve the reproducibility of the work that we publish. This form provides structure for consistency and transparency in reporting. For further information on Nature Research policies, see Authors & Referees and the Editorial Policy Checklist.

Statistics

For all statistical analyses, confirm that the following items are present in the figure legend, table legend, main text, or Methods section.

n/a	Confirmed	
☐	☒	The exact sample size (*n*) for each experimental group/condition, given as a discrete number and unit of measurement
☐	☒	A statement on whether measurements were taken from distinct samples or whether the same sample was measured repeatedly
☐	☒	The statistical test(s) used AND whether they are one- or two-sided<br>*Only common tests should be described solely by name; describe more complex techniques in the Methods section.*
☐	☒	A description of all covariates tested
☐	☒	A description of any assumptions or corrections, such as tests of normality and adjustment for multiple comparisons
☐	☒	A full description of the statistical parameters including central tendency (e.g. means) or other basic estimates (e.g. regression coefficient) AND variation (e.g. standard deviation) or associated estimates of uncertainty (e.g. confidence intervals)
☐	☒	For null hypothesis testing, the test statistic (e.g. *F*, *t*, *r*) with confidence intervals, effect sizes, degrees of freedom and *P* value noted<br>*Give P values as exact values whenever suitable.*
☒	☐	For Bayesian analysis, information on the choice of priors and Markov chain Monte Carlo settings
☒	☐	For hierarchical and complex designs, identification of the appropriate level for tests and full reporting of outcomes
	☒	Estimates of effect sizes (e.g. Cohen's *d*, Pearson's *r*), indicating how they were calculated

Our web collection on statistics for biologists contains articles on many of the points above.

Software and code

Policy information about availability of computer code

Data collection	Cell suspensions were processed for single-cell RNA-sequencing using Chromium Single Cell 3' Library and Gel Bead Kit v2 (10X Genomics, PN-120237) according to 10X Genomics guidelines. Libraries were sequenced on an Illumnia NextSeq 500 using 150 cycles high output V2 kit (Read 1-26, Read2-98 and Index 1-8 bases). The Cell Ranger package (v3.0.2) was used to align high quality reads to the mm10 transcriptome (quality control reports available: https://stanford.io/37sXZV3).
Data analysis	Code for the algorithms used in this manuscript is publicly available:<br><br>Variable gene selection, dimensional reduction and clustering was performed using Seurat version 2.3:<br><br>https://github.com/satijalab/seurat<br><br>Data imputation was performed using a customized implementation of MAGIC:<br><br>https://github.com/kbrulois/magicBatch<br><br>Trajectory Analysis was performed using the tSpace algorithm:<br><br>https://github.com/hylasD/tSpace

For manuscripts utilizing custom algorithms or software that are central to the research but not yet described in published literature, software must be made available to editors/reviewers. We strongly encourage code deposition in a community repository (e.g. GitHub). See the Nature Research guidelines for submitting code & software for further information.

Data

Policy information about availability of data

All manuscripts must include a data availability statement. This statement should provide the following information, where applicable:

- Accession codes, unique identifiers, or web links for publicly available datasets
- A list of figures that have associated raw data
- A description of any restrictions on data availability

Raw single cell RNAseq data were deposited at the Gene Expression Ominibus (GEO) under accession number GSE140348 [https://www.ncbi.nlm.nih.gov/geo/query/acc.cgi?acc=GSE140348]. Source data for individual figures will be made available upon request.

Field-specific reporting

Please select the one below that is the best fit for your research. If you are not sure, read the appropriate sections before making your selection.

☒ Life sciences ☐ Behavioural & social sciences ☐ Ecological, evolutionary & environmental sciences

For a reference copy of the document with all sections, see nature.com/documents/nr-reporting-summary-flat.pdf

Life sciences study design

All studies must disclose on these points even when the disclosure is negative.

Sample size	No method was used to pre-determine sample size.
Data exclusions	Low quality cells and contaminating cell types such as lymphocytes were excluded. See methods for complete description of exclusion criteria.
Replication	Single cell analysis was performed sucessfully using 3 independent cohorts of mice.
Randomization	Randomization was not relevant to this study because it did not involve
Blinding	Blinding was used for certain experiments to

Reporting for specific materials, systems and methods

We require information from authors about some types of materials, experimental systems and methods used in many studies. Here, indicate whether each material, system or method listed is relevant to your study. If you are not sure if a list item applies to your research, read the appropriate section before selecting a response.

Materials & experimental systems

n/a	Involved in the study	
☐	☒	Antibodies
☒	☐	Eukaryotic cell lines
☒	☐	Palaeontology
☐	☒	Animals and other organisms
☒	☐	Human research participants
☒	☐	Clinical data

Methods

n/a	Involved in the study	
☒	☐	ChIP-seq
☐	☒	Flow cytometry
☒	☐	MRI-based neuroimaging

Antibodies

Antibodies used	***see below***
Validation	***Antibodies were validated by flow cytometry or IHC as needed.***

The following antibodies (clone; catalogue number; dilution) were used for both microscopy and FACS: Brilliant Violet (BV) 605-conjugated CD31 (390; 102427; 1:100), peridinin chlorophyll protein–cyanine 5.5–conjugated anti-CD45 (30-F11; 103131; 1:100), peridinin chlorophyll protein–cyanine 5.5–conjugated anti-Ter-119 (TER-119 TER-119; 116227; 1:100), peridinin chlorophyll protein–cyanine 5.5–conjugated anti-CD11a (H155-78; 141007; 1:100), peridinin chlorophyll protein–cyanine 5.5–conjugated anti-CD326 (G8.8; 118219; 1:100), phycoerythrin-cyanine 7-conjugated anti-Gp38 (8.1.1; 127411; 1:100), anti-VE-Cadherin (VECD1; 138101), and BV421-conjugated anti-CXCR4 (L276F12; 146511) were from Biolegend. BV421-conjugated anti-CD146 (ME-9F1; 740095; 1:100) and BV480 Streptavidin (564876; 1:200) were from BD Biosciences. Anti-estrogen receptor alpha antibody (SP1; ab16660; 1:100) and Anti-ERG antibody (EPR3864; ab196149 and ab196374) were from Abcam. Anti-CD276 (MIH35; 16-5937-81) was from Thermo Fisher Scientific. Anti-PNAd (MECA-79), anti-Ly6c (Monts1), anti-EMCN (5C7), anti-PODXL (MECA-99), anti-ICAM-1 (BE29G1), anti-VCAM-1 (6C2.1), anti-PLVAP (MECA-32) and anti-Slex (F2) were produced in-house from hybridomas; labelled with DyLight Antibody Labeling Kits or Biotin labeling kit (Thermo Fisher Scientific), and used at a concentration of 10ug/ml. Alexa Fluor 488–conjugated donkey antibody to rabbit IgG (711-546-152; 1:500) was from Jackson ImmunoResearch Laboratories. Antibodies for in vivo blockade of selectins were from BD Biosciences: Anti-E-selectin (10E9.6; 553749), anti-P-selectin (RB40.34; 550289), and NA/LE isotype control rat IgG1 (R3-34; 554682). Antibody dilutions for FACS are approximate; actual dilution varied between experiments.

Animals and other organisms

Policy information about studies involving animals; ARRIVE guidelines recommended for reporting animal research

Laboratory animals	See methods for complete description
Wild animals	No wild animals were used in this study
Field-collected samples	No field samples were used in this study
Ethics oversight	Institutional Animal Care and Use Committee (IACUC)

Note that full information on the approval of the study protocol must also be provided in the manuscript.

Flow Cytometry

Plots

Confirm that:

☒ The axis labels state the marker and fluorochrome used (e.g. CD4-FITC).

☒ The axis scales are clearly visible. Include numbers along axes only for bottom left plot of group (a 'group' is an analysis of identical markers).

☒ All plots are contour plots with outliers or pseudocolor plots.

☒ A numerical value for number of cells or percentage (with statistics) is provided.

Methodology

Sample preparation	See methods for complete description
Instrument	Aria III
Software	Data were collected using FACSDiva and analyzed using FlowJo (v10.3).
Cell population abundance	Blood endothelial cells comprised about 1% of the cell suspension used for sorting
Gating strategy	A figure exemplifying the gating strategy is provided in Figure 1A

☐ Tick this box to confirm that a figure exemplifying the gating strategy is provided in the Supplementary Information.
