# Supplementary material for: A molecular map of murine lymph node blood vascular endothelium at single cell resolution
Source: Nat Commun. 2020 Jul 30;11:3798. doi: 10.1038/s41467-020-17291-5 (PMC7393069; doi:10.1038/s41467-020-17291-5)
Supplement: Supplementary file 5 — Description of Additional Supplementary Files [file 41467_2020_17291_MOESM5_ESM.pdf]

Title: Supplementary Data 1 Differential gene expression analysis of individual subsets

Description: Each sheet contains fold-change values (log2) and p-values (-log10) for the indicated subset (sheet name) compared to other BEC. Each differential gene expression analysis is appended with additional columns corresponding to the average normalized counts for each gene in each subset.

Title: Supplementary Data 2 Conservation of significantly upregulated genes among lymphoid and non-lymphoid CRP and CRP-like cells

Description: CRP-like cells were identified in several tissues: peripheral lymph node, PLN; bone marrow endothelial cells, BMEC; Fat; Heart_and_Aorta; Limb_Muscle; Lung; Mammary_Gland; Trachea. Within each of these tissues, CRP were compared with other cells using a zero-inflated negative binomial model followed by likelihood-ratio test (log2FC and p-values: columns B through Y). As a control for specificity, Art were also compared with other cells in each tissue in the same way (columns Z through AW). Genes with a log2FC > 0.4 and p-value < 0.01 in a given sample were considered "up" and are marked with a 1 and colored rose (columns BA through BK, CRP; and BO through BY, Art). Genes that are up in multiple samples were identified using various criteria to select for different degrees of conservation: "low_conservation", up in at least 2 lymphoid samples and 2 non-lymphoid samples; "moderate_conservation", up in at least 2 lymphoid samples and 3 non-lymphoid samples; "high_conservation", up in at least 2 lymphoid samples and 4 non-lymphoid samples (columns AX through AZ, CRP; and columns BL through BN, Art). Filters (accessed by clicking the down arrow in excel) can be applied separately or in combination to display differentially expressed genes for individual tissues, genes conserved at various degrees across tissues or genes that are specifically upregulated in CRP (or Art).
